# Integrative Characterization of the Role of IL27 In Melanoma Using Bioinformatics Analysis

**DOI:** 10.3389/fimmu.2021.713001

**Published:** 2021-10-18

**Authors:** Chunyu Dong, Dan Dang, Xuesong Zhao, Yuanyuan Wang, Zhijun Wang, Chuan Zhang

**Affiliations:** ^1^ Department of Pediatric Surgery, The First Hospital of Jilin University, Changchun, China; ^2^ Department of Neonatology, The First Hospital of Jilin University, Changchun, China; ^3^ Department of Pediatric Ultrasound, The First Hospital of Jilin University, Changchun, China

**Keywords:** *IL27*, CD8^+^ T cells, β-catenin, immunotherapy, melanoma, tumor microenvironment

## Abstract

**Background:**

*IL27* has been reported to play dual roles in cancer; however, its effects on the tumor microenvironment (TME), immunotherapy, and prognosis in melanoma remain largely unclear. This study was aimed to uncover the effects of *IL27* on TME, immunotherapy and prognosis in patients with melanoma.

**Methods:**

RNA-seq data, drug sensitivity data, and clinical data were obtained from TCGA, GEO, CCLE, and CTRP. Log-rank test was used to determine the survival value of *IL27*. Univariate and multivariate Cox regression analyses were employed to determine the independent predictors of survival outcomes. DAVID and GSEA were used to perform gene set functional annotations. ssGSEA was used to explore the association between *IL27* and immune infiltrates. ConsensusClusterPlus was used to classify melanoma tissues into hot tumors or cold tumors.

**Results:**

Clinically, *IL27* was negatively correlated with Breslow depth (*P* = 0.00042) and positively associated with response to radiotherapy (*P* = 0.038). High *IL27* expression showed an improved survival outcome (*P* = 0.00016), and could serve as an independent predictor of survival outcomes (hazard ratio: 0.32 - 0.88, *P* = 0.015). Functionally, elevated *IL27* expression could induce an enhanced immune response and pyroptosis (*R* = 0.64, *P* = 1.2e-55), autophagy (*R* = 0.37, *P* = 7.1e-17) and apoptosis (*R* = 0.47, *P* = 1.1e-27) in patients with melanoma. Mechanistically, elevated *IL27* expression was positively correlated with cytotoxic cytokines (including *INFG* and *GZMB*), enhanced immune infiltrates, and elevated CD8/Treg ratio (*R* = 0.14, *P* = 0.02), possibly driving CD8^+^ T cell infiltration by suppressing β-catenin signaling in the TME. Furthermore, *IL27* was significantly associated with hot tumor state, multiple predictors of response to immunotherapy, and improved drug response in patients with melanoma.

**Conclusions:**

*IL27* was correlated with enriched CD8^+^ T cells, desirable therapeutic response and improved prognosis. It thus can be utilized as a promising modulator in the development of cytokine-based immunotherapy for melanoma.

## Introduction

Skin melanoma is a fatal type of cutaneous carcinoma (1), and the incidence of melanoma has been increasing annually (2, 3). Despite impressive advances in immune and targeted therapies (4, 5), approximately half of melanoma patients will develop intrinsic or acquired resistance to immunotherapy (6–9), with a five-year survival rates of 26% to 66% for advanced melanoma based on the statistics of the American Cancer Center. Given the plight of therapeutic resistance, there is an urgent need to uncover the mechanisms of resistance to immunotherapy.

Accumulating evidence suggests that the tumor microenvironment (TME) plays an important role in tumor progression. Solid tumors can be classified into immunologically hot tumors and cold tumors; hot tumors are responsive to cancer immunotherapy, whereas cold tumors are refractory to the treatment (10, 11). Immunologically cold tumors are characterized by low mutation burden, low infiltration of cytotoxic immune cells and high abundance of myeloid-derived suppressor cells, resulting in worse clinical responses to immune checkpoint blockade (ICB) (12–14). Nonetheless, preliminary studies have shown that it is possible to turn cold tumors into hot tumors (10, 15). Therefore, it is crucial to uncover the comprehensive mechanism underlying immunologically cold tumors, which would help in developing a strategy for turning cold tumors into hot tumors.

*IL27* is an immunomodulatory cytokine that plays pleiotropic roles in the context of tumor immune environment (TME). *IL27* is reported as a protumor cytokine in pancreatic cancer and hepatocellular carcinoma (16, 17), whereas considered as an antitumor factor in lung cancer and melanoma (18, 19). Meanwhile, *IL27* is known to exert dual roles in the TME, as it can induce effector immune response as well as stimulate tumor expansion by suppressing immune function (20). However, the association between *IL27* and TME and immunotherapy is currently largely unclear.

To date, very limited research has been carried out regarding the relationship between *IL27* and melanoma. Although early literature shows that *IL27* may have potential anti-melanoma effects by promoting the activity of CD8^+^ T cells (21, 22), there is still a lack of specific molecular mechanisms underlying the impact of *IL27* on CD8^+^ T cells. Moreover, whether *IL27* could serve as a predicting biomarker for survival and response to immunotherapy is unknown. Furthermore, the mechanisms of the contradictory effect of *IL27* on tumors remains elusive.

Given the controversial roles of *IL27*, this study aimed to clarify the association of *IL27* with prognosis, TME, and immunotherapy in melanoma. Our findings would contribute to uncovering the multifaceted roles of *IL27* and provide evidence for future cytokine-based immunotherapy against melanoma.

## Materials and Methods

### Data Acquisition

RNA-seq data and clinical data of 470 melanoma patients (including 472 tissue samples) were obtained from the Cancer Genome Atlas (TCGA) cohort. Clinical data included age, gender, clinical stage, tumor status (with tumor or tumor free), Breslow depth, Clark level, pathological stage, response to radiotherapy, survival time, and survival status. Tumor status is one of the clinical characteristics of melanoma patients in the TCGA cohort.

Five melanoma cohorts (GSE133713, GSE50509, GSE65904, GSE22155, and GSE19234) with RNA-seq data for *IL27* and corresponding survival materials were used to validate the survival significance of *IL27*. Moreover, we merged these five GEO datasets by removing the batch effect and generated a larger combined cohort to further validate the survival value of *IL27* in patients with melanoma.

RNA-seq data of 214 melanoma patients from GSE65904 were used as an independent dataset to verify the main results generated from RNA-seq data from the TCGA cohort.

RNA-seq profiles, including RNA-seq data from control mice and *IL27* overexpressing mice treated intramuscularly with plasmids containing *IL27*, were obtained from GSE178142 (23), which was utilized to investigate the effect of overexpressed *IL27* on the biological behavior of tumors *in vivo*.

RNA-seq data of 21 melanoma cell lines were acquired from the Cancer Cell Line Encyclopedia (CCLE) (24, 25), which profiles gene expression in cancer cells. Drug response data were available from the Cancer Therapeutics Response Portal (CTRP) (26), which characterizes the response of cancer cell lines to a vast spectrum of therapeutic agents.

### DAVID

Gene annotation was performed using the Database for Annotation, Visualization, and Integrated Discovery (DAVID, v6.8) (27), which allows us to investigate the biological functions and signaling pathways a given gene set is involved in. Gene annotation included Gene Oncology (GO) and Kyoto Encyclopedia of Genes and Genomes (KEGG) pathway analyses. GO comprised of three independent categories: biological process (BP), molecular function (MF), and cellular component (CC). Terms with FDR < 0.05 were considered as significantly enriched.

### GSEA

To confirm the findings obtained using DAVID, we performed gene set enrichment analysis (GSEA, v3.0) (28) using TPM of RNA-seq data for 470 melanoma patients from the TCGA cohort. GSEA is a computational approach that determines whether *a priori* defined gene set shows statistically significant, concordant differences between two biological states (e.g., phenotypes). The key parameters were set as follows: the number of permutations at 1000, weighted enrichment statistic, metric for ranking genes (Signal2Noise), max size (500), and min size (15). The selection criteria included FDR < 0.05 and |NES| > 1.

### Differential expression analysis

We performed differentially expressed genes (DEGs) analysis based on RNA-seq data using R package edgeR (29), which implements a series of statistical methods including empirical Bayes estimation, exact tests, generalized linear models, and quasi-likelihood tests. Selection criteria for DEGs were as follows: |logFC| > 2 and FDR < 0.01.

### ssGSEA

To investigate the association of *IL27* with certain phenotypes, we performed ssGSEA analysis using R package “GSVA” (30). Gene set variation analysis (GSVA) is a non-parametric, unsupervised method to calculate variation of gene set enrichment through the samples from an expression dataset. Each ssGSEA enrichment score represents the degree to which the genes in a particular gene set are coordinately up- or down-regulated within a sample. The key parameters were as follows: kcdf = “Gaussian”, min.sz = 1, max.sz = Inf, tau = 0.25, abs.ranking = TRUE. The gene sets for pyroptosis, apoptosis, autophagy, and β-catenin signaling were obtained by retrieving previous literature and are provided in **Supplementary Table S1**.

### TIMER

We reexamined the association between *IL27* expression and CD8^+^ T cell abundance using the Tumor Immune Estimation Resource (TIMER 2.0) (31). TIMER is a comprehensive resource for assessing the clinical relevance of tumor-immune infiltrations, which can also characterize the association between genes and tumor-infiltrating immune cells across diverse types of cancer.

### Unsupervised Clustering

Unsupervised clustering was implemented to classify melanoma tissues into hot or cold tumors. We performed unsupervised clustering using R package “ConsensusClusterPlus”, which is based on a computational method called consensus clustering (32). Consensus clustering can provide quantitative evidence for determining the number of potential clusters within the RNA-seq data. Here, we used RNA-seq data from 472 melanoma samples from the TCGA cohort as input. The key operating parameters included 80% item resampling, a maximum evaluated k of 20, and 1000 repetitions.

### Statistics

Statistical analyses were performed using R software (Version 4.0.1). The normal distribution of continuous variables was assessed using the Shapiro-Wilk test, and the homogeneity of variance was assessed using Bartlett’s test. The independent sample t-test or Wilcoxon signed rank test was used based on the data homogeneity of variance and normal distribution. Survival analysis was performed using the log-rank test. Pearson’s correlation coefficients were computed to determine the correlation between two continuous variables. The correlation intensities were classified into five grades according to the absolute value of the correlation coefficient: 0.00-0.19 corresponded to very weak, 0.20-0.39 corresponded to weak, 0.40-0.59 corresponded to moderate, 0.60-0.79 corresponded to strong, and 0.80–1.0 to very strong (33). *P* < 0.05 was considered significant.

## Results

### Clinical Significance of *IL27* in Melanoma

**Figure 1:**
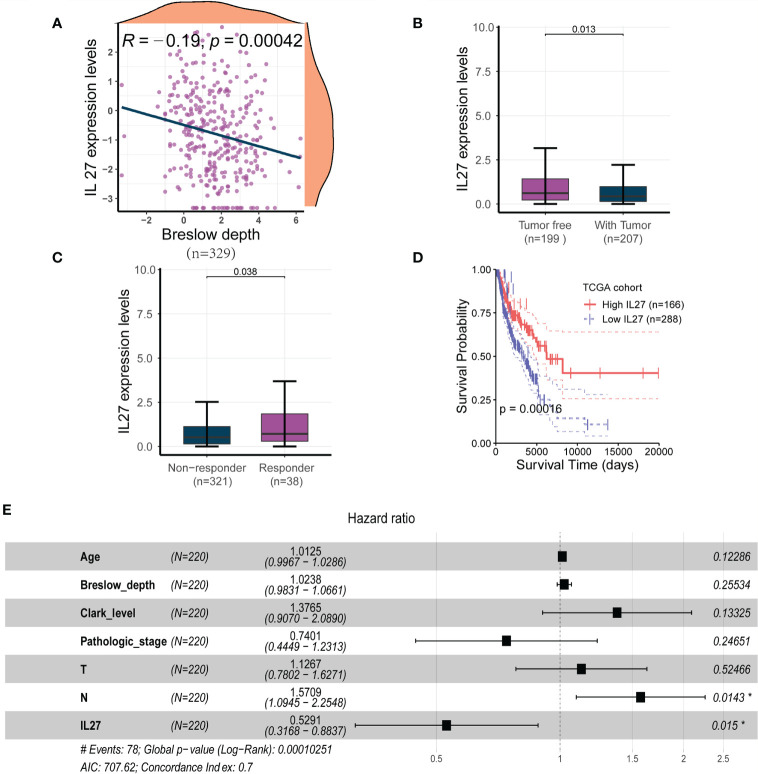
*IL27* was an independent predictor for improved survival in melanoma **(A)** *IL27* expression was negatively correlated with Breslow depth, suggesting its potential implication in the progression of tumor. **(B)** *IL27* was highly expressed in patients without tumor than those with tumor, further underscoring its antitumor effects on tumor growth. **(C)** *IL27* was markedly higher in patients who responded to radiation therapy than those who were resistant to radiation therapy, suggesting *IL27* may be involved in response to melanoma therapy. **(D)** High *IL27* expression conferred a survival advantage in melanoma patients. **(E)** Multivariate Cox regression analysis showed *IL27* could serve as an independent predictor of favorable survival outcomes. *indicates statistical significance.

To investigate the clinical relevance of *IL27*, we analyzed RNA-seq data and clinical information from the TCGA cohort of 470 patients with melanoma. We found that *IL27* expression was inversely correlated with Breslow depth (Pearson correlation test; *R* = - 0.19, *P* = 0.00042; **Figure 1A**) in 329 patients with complete Breslow depth value, and was significantly overexpressed in patients with tumor than in patients without tumor (*t*-test; *P* = 0.013; **Figure 1B**). Moreover, *IL27* was markedly higher in 38 patients who benefited from radiotherapy than that in 321 counterparts who resisted it (*t*-test; *P* = 0.038; **Figure 1C**), suggesting that *IL27* may be linked to response to melanoma therapy.

Since *IL27* was shown to have a critical clinical relevance, we sought to investigate its survival value. First, we performed a survival analysis for *IL27*, and found that high expression of *IL27* was linked to better survival than its low expression (log-rank test; *P* = 0.00016; **Figure 1D**). Next, we wondered whether *IL27* could serve as an independent predictor of survival in melanoma. We performed the univariate Cox regression analysis using gender, age, Breslow depth, Clark level, ulceration, pathologic stages, T, N, M, and *IL27* expression as inputs; our analysis showed that age, Breslow depth, Clark level, pathologic stage, T, N and *IL27* expression were significantly associated with the survival outcomes (*P* < 0.05; **Supplementary Table S1**). We then performed the multivariate Cox regression analysis using these parameters as inputs; the results showed that *IL27* was an independent predictor of improved survival outcomes (hazard ratio: 0.32 - 0.88, *P* = 0.015; **Figure 1E**).

### Validation of the Prognostic Value of *IL27* in Multiple Cohorts

**Figure 2:**
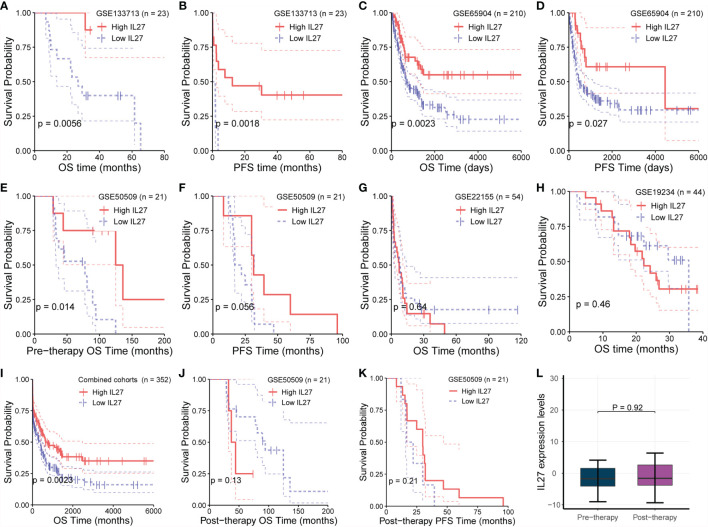
Validation of prognostic value of IL27 in multiple cohorts. **(A, B)** High expression of *IL27* can reflect improved progression-free survival (PFS) and overall survival (OS) in GSE133713. **(C, D)** High expression of *IL27* can reflect improved PFS and OS in GSE65904. **(E, F)** High expression of *IL27* can reflect improved PFS and OS in GSE50509. **(G, H)** *IL27* has no prognostic value in GSE22155 and GSE19234. **(I)** High expression of *IL27* can reflect improved OS in the combined cohort (including patients from GSE133713, GSE65904, GSE22155, GSE19234, and GSE50509). **(J, K)** *IL27* expression after medication had no significant prognostic significance in GSE50509. **(L)** *IL27* expression did not change after medication in GSE50509.

Although we demonstrated that *IL27* is predictive of favorable survival in the TCGA cohort, we wondered if the prognostic value of *IL* 27 was also valid in other melanoma cohorts. To investigate the prognostic value of *IL27* in different cohorts, we investigated its prognostic relevance in five melanoma cohorts (viz. GSE133713, GSE65904, GSE22155, GSE19234, and GSE50509) using the log-rank test. We observed that high expression of *IL27* reflected improved progression-free survival (PFS; **Figures 2B, D, F**) and overall survival (OS; **Figures 2A, C, E**) in three cohorts, namely GSE133713, GSE65904, and GSE50509, as well as in the combined cohort (i.e., considering patients from GSE133713, GSE65904, GSE22155, GSE19234, and GSE50509; **Figure 2I**), whereas *IL27* had no prognostic value in GSE22155 and GSE19234 cohorts (**Figures 2G, H**).

Given these contradictory results, we analyzed the characteristics of the populations from GSE22155 and GSE19234 cohorts, and found that the enrolled patients in these two cohorts had all stage III/IV melanoma. Specifically, the patients in GSE19234 underwent surgery twice at different time points: the first surgery was done to remove the primary tumor when melanoma was diagnosed, and the second surgery was carried out to remove metastatic tumor when melanoma metastasized. In other words, the samples used for RNA sequencing were collected when the patient relapsed. Therefore, it is reasonable to speculate that these relapsed patients had undergone drug treatment and developed primary or secondary drug resistance, leading to relapse. When drug resistance occurs, the downstream effects of *IL27* are blocked or weakened, while the expression of *IL27* may continue to increase due to the negative feedback mechanism as the tumor develops, which could cause a phenomenon in which high expression of *IL27* may not have any prognostic value or may be associated with worse survival in resistant patients due to the downstream effects of *IL27* being blocked or weakened.

To confirm the hypothesis that the effect of *IL27* could be offset by drug resistance following medication, we analyzed its prognostic value using RNA-seq data and survival data of melanoma patients from GSE50509 cohort who had received dabrafenib or vemurafenib treatment. Of note, in GSE50509, all enrolled patients underwent RNA sequencing before and after dabrafenib or vemurafenib treatment, respectively, and had corresponding survival data. As expected, *IL27* expression after medication had no significant prognostic significance, and even the prognosis of people with high *IL27* expression was slightly poor in patients treated with dabrafenib or vemurafenib (**Figures 2J, K**), whereas while high expression of *IL27* can reflect improved progression-free survival and overall survival (**Figures 2E, F**). Notably, we found that *IL27* expression did not change after the treatment (**Figure 2L**), suggesting that medication did not influence the expression of *IL27*, but might have affected its downstream effects. These results suggest that the prognostic value of *IL27* is related to its own expression, and could be offset by drug resistance following medication. Further, these findings may explain the contradictory effects of *IL27* observed in cancers.

### Functional Annotation of *IL27*

**Figure 3:**
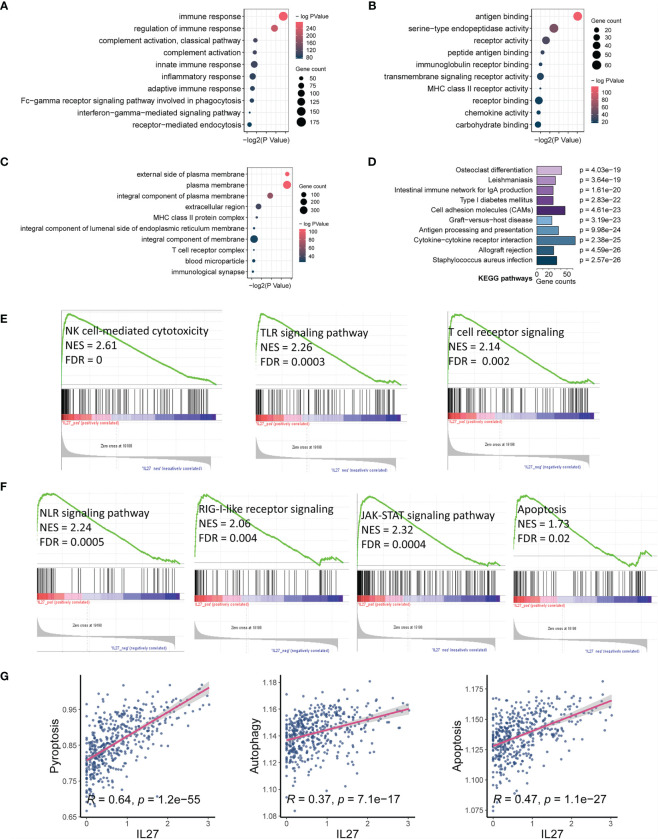
*IL27* expression was markedly associated with tumor immunity and apoptosis. **(A–C)** Bubble plots displayed the top 10 BP, MF and CC terms that were significantly associated with *IL27*, and they were all immune-related. **(D)** Bar plot showed the top 10 KEGG terms that were significantly correlated with *IL27*, and they were also immune-related. **(E)** The results of GSEA showed three *IL27*-related signaling pathways, which were all related to activation of cytotoxic immune cells. **(F)** The results of GSEA showed four *IL27*-related signaling pathways, which were all related to cell pyroptosis and apoptosis. **(G)** *IL27* expression was positively associated with pyroptosis, autophagy and apoptosis based on ssGSEA.

As we found that *IL27* was implicated in the progression and prognosis of melanoma, we next sought to investigate the biological function of *IL27*. First, we performed a correlation analysis between *IL27* and other genes using RNA-seq data of melanoma patients from the TCGA cohort. The results showed that there were 1047 genes significantly associated with *IL27* (*P* < 0.01, |*R*| > 0.4; **Supplementary Table S2**). Second, we performed DAVID using these 1047 genes, and obtained 184 enriched BP terms, 42 MF terms, 33 enriched CC terms and 48 KEGG terms (FDR < 0.05; **Supplementary Tables S3**). We found that all biological functions and signaling pathways were all immune-related (**Figures 3A–D**), strongly implying that *IL27* is involved in the TME.

To further assess the effects of IL27 on signaling pathways, we performed GSEA using RNA-seq data of 472 melanoma samples from the TCGA cohort, and obtained 30 positively correlated KEGG pathways (FDR < 0.05, NES > 1; **Supplementary Table S4**), including natural killer cell-mediated cytotoxicity, Toll-like receptor signaling pathway, T cell receptor signaling pathway, NOD-like receptor signaling pathway, RIG-I-like receptor signaling pathway, JAK-STAT signaling pathway, and apoptosis (**Figures 3E, F**). To verify the reliability of the results obtained from functional annotation, the same analysis process was used to assess another independent dataset (GSE65904). The results from this analysis also demonstrated that *IL27* was primarily involved in the immune response, with multiple overlapping gene oncology and pathways within the results obtained for both TCGA and GSE65904 (**Supplementary Figures S1A–D**).

We noticed that the signaling pathways associated with *IL27* consisted of NLR signaling pathway, TLR signaling pathway, T cell receptor signaling pathway, RIG-I-like receptor signaling pathway, JAK-STAT signaling pathway, and apoptosis. Among them, the NLR signaling pathway (34), TLR signaling pathway (35), and T cell receptor signaling pathway (36, 37) have been reported to be correlated with enhanced immune response, which is in line with our findings above. The RIG-I-like receptor signaling pathway is known to be implicated in apoptosis through the JAK-STAT signaling pathway (38, 39), which seems to be contrary to enhanced immunity. Considering that bulk RNA sequencing measures the mRNA expression of the entire tumor tissue, which includes tumor cells, stromal cells, immune cells, and some extracellular cytokines, we speculated that *IL27* could promote programmed cell death of tumor cells by enhancing effector immune cells. In effect, NOD-like receptor signaling pathway has also been reported to correlate with pyroptosis (40). Consistent with our speculation that programmed cell death occurs in tumor cells, we have previously demonstrated that high *IL27* expression indeed is associated with elevated immune response and improved survival in melanoma. We next investigated the effects of *IL27* expression on programmed cell death, including pyroptosis, autophagy and apoptosis, using ssGSEA. As expected, *IL27* expression was markedly correlated with pyroptosis (Pearson correlation test; *R* = 0.64, *P* = 1.2e-55), autophagy (Pearson correlation test; *R* = 0.37, *P* = 7.1e-17), and apoptosis (Pearson correlation test; *R* = 0.47, *P* = 1.1e-27) (**Figure 3G**). We used the same analysis process for GSE65904, and found that *IL27* was also significantly correlated with pyroptosis in this dataset as well (Pearson correlation test; *R* = 0.18, *P* = 0.008; **Supplementary Figure S1D**).

### Validation of Biological Function of *IL27*

**Figure 4:**
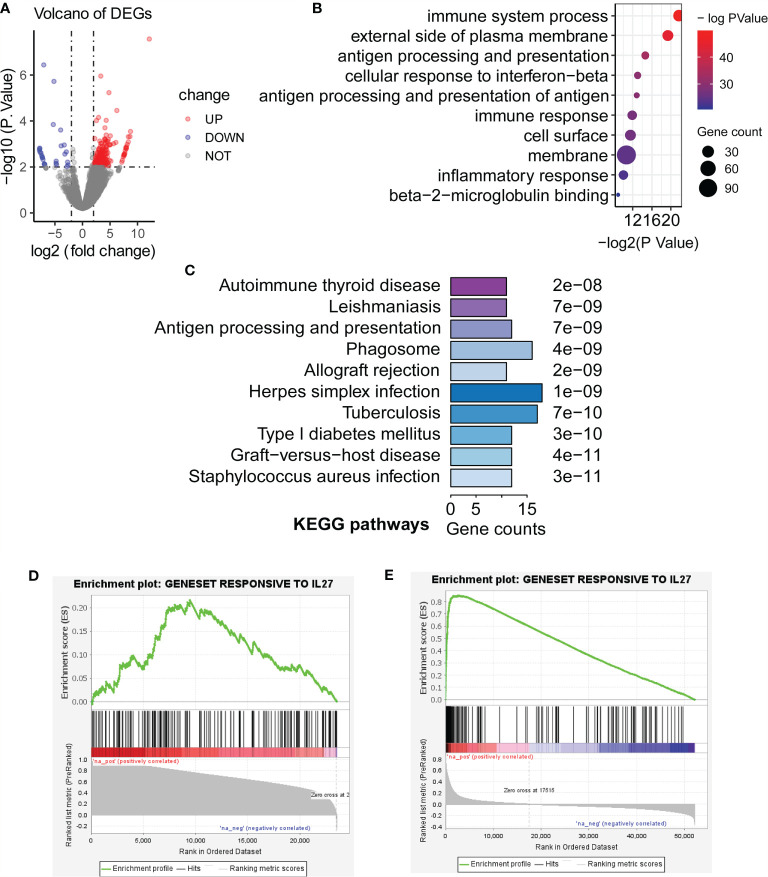
Validation of biological function of *IL27*. **(A)** Differentially expressed genes (DEGs) were computed between control mice and *IL27* overexpressing mice (Blue dots represent downregulated DEGs, and red dots represent upregulated DEGs; |logFC| > 2 and *P* < 0.01). **(B, C)** Upregulated genes in response to *IL27* were mainly enriched in immune-related biological processes and keg pathways (FDR < 0.05). **(D)** Upregulated genes in response to *IL27* treatment were positively co-expressed with *IL27* in human prostate cancer (GSE32448; FDR = 0.014, NES = 1.47). **(E)** Upregulated genes in response to *IL27* treatment were positively co-expressed with *IL27* in human melanoma cancer (TCGA-SKCM; FDR = 0.000, NES = 3.33).

Although we have observed that *IL27* was linked to enhanced immune response as described above, it remained unclear whether *IL27* was a driver of the immune response or simply a passenger. To further validate the effects of *IL27* on the biological function of tumors *in vivo*, we searched and found an ideal dataset (GSE178142) (23), including RNA-seq data from control mice (GSM5380810 and GSM5380811) and *IL27* overexpressing mice treated intramuscularly with plasmids containing *IL27* (GSM5380806 and GSM5380807). We downloaded RNA-seq data for control mice and experimental mice, and converted the ensemble ID to gene symbol. Differentially expressed genes (DEGs) were computed between control mice and *IL27* overexpressing mice using R package “edgeR”. A total of 233 DEGs, including 207 upregulated DEGs and 26 downregulated DEGs, were obtained (|logFC| > 2 and *P* < 0.01; **Figure 4A**).

Next, we next performed GO and KEGG analysis based on 207 upregulated DEGs in *IL27* overexpressing mice compared with control mice using DAVID. Consistent with the findings in the previous step, upregulated genes in response to *IL27* were mainly enriched in immune-related biological processes and keg pathways (FDR < 0.05; **Figures 4B, C**).

To further validate the effects of *IL27* on immune response in human cancers, we investigate whether upregulated genes in response to *IL27* treatment will be positively co-expressed with *IL27* in human prostate cancer (GSE32448) and melanoma cancer (the TCGA-SKCM cohort). We first defined a gene set (gmt file) for the 207 genes up-regulated in response to *IL27* using RNA-Seq data from GSE178142. Then, we used the expression data of the GSE32448 cohort and TCGA cohort, respectively, to define a rnk file for all expressed genes based on their co-expression with IL27. Finally, we applied GSEAPreranked analysis of the rnk file to against the gmt file. The expectation is that upregulated genes in response to *IL27* treatment will be positively co-expressed with *IL27*. As expected, upregulated genes in response to *IL27* treatment were positively co-expressed with *IL27* in both human prostate cancer (GSE32448; FDR = 0.014, NES = 1.47; **Figure 4D**) and human melanoma cancer (TCGA-SKCM; FDR = 0.000, NES = 3.33; **Figure 4E**), further supporting *IL27* as a driver gene. Moreover, tumor volume for the control group was observed to be significantly larger than that in *IL27* overexpressing group in a previously reported study (23). Altogether, these combined analyses suggest that *IL27* acts as a driver gene and has an anti-tumor effect.

### Association of *IL27* Expression With TME

**Figure 5:**
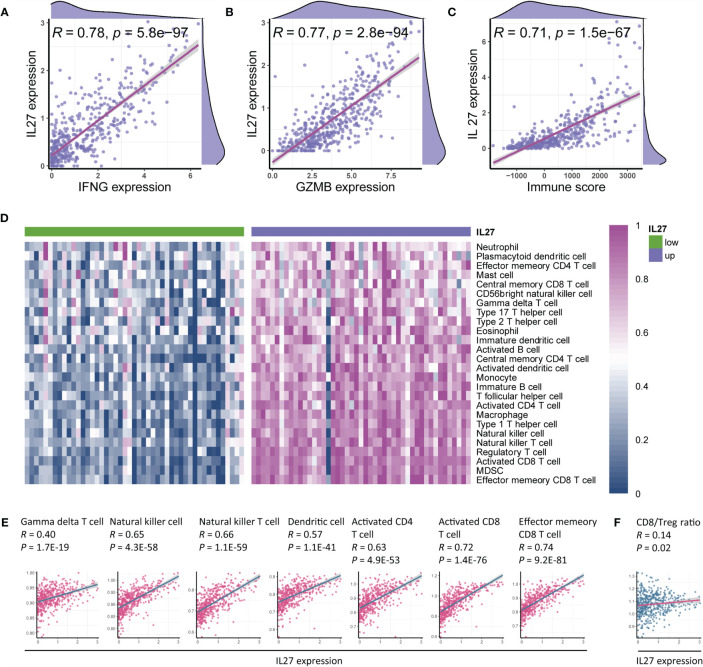
*IL27* was associated with antitumor immunity and therapeutic efficacy of immunotherapy. **(A–C)** IL27 was markedly positively correlated with IFNG, GZMB, and immune score, suggesting its role in antitumor immunity (Pearman’s correlation test). **(D)** Immune cells were enriched in patients with high expression of *IL27*, implying its function of promoting immunity. **(E)** *IL27* was markedly associated with antitumor immune cells, including gamma delta T cell, natural killer cell, natural killer T cell, dendritic cell, activated CD4^+^ T cell, activated CD8^+^ T cell, and effector memory CD8^+^ T cell (Pearman’s correlation test). **(F)** *IL27* was positively correlated with CD8/Treg ratio, suggesting *IL27* as a potential predictor of response to immunotherapy (Pearman’s correlation test).

As the findings of functional annotation indicated that *IL27* was involved in TME, we further investigated the association of *IL27* with TME. We first analyzed the association of *IL27* with *IFNG*, *GZMB*, and immune score, which are known to be associated with antitumor immunity (41, 42). Surprisingly, we observed a marked correlation between *IL27* and *IFNG*, *GZMB*, and immune score (Pearson correlation test; *R* = 0.8, *R* = 0.77, and *R* = 0.71, respectively; **Figures 5A–C**), suggesting its role in the antitumor immunity. To verify the reliability of the results, we also used the same analysis process for GSE65904 cohort, and found that *IL27* was also significantly correlated with *IFNG*, *GZMB*, and immune score in this dataset as well (Pearson correlation test; *R* = 0.34, *R* = 0.28, *R* = 0.23, respectively; **Supplementary Figures S2A–C**).

To comprehensively characterize the effects of *IL27* expression on immune cells, we first estimated the abundance of each immune cell using ssGSEA based on RNA-seq data from the TCGA cohort of melanoma patients (**Supplementary Table S5**). We next compared the abundance of immune cells between patients with high *IL27* expression and those with low *IL27* expression, and found that immune cells were markedly enriched in patients with high *IL27* expression (**Figure 5D**), suggesting its function in the promotion of immunity.

To further validate the relationship between *IL27* and antitumor immunity, we performed a correlation analysis between *IL27* and antitumor immune cells, including gamma delta T cells, natural killer cells, natural killer T cells, dendritic cells, activated CD4^+^ T cells, activated CD8^+^ T cells, and effector memory CD8^+^ T cells. Intriguingly, *IL27* was found to be significantly positively correlated with the levels of these immune cells (Pearson correlation test; *R* ranging from 0.4 to 0.74, *P* < 0.0001; **Figure 5E**), thus further indicating the antitumor function of *IL27* in TME. We then used the same analysis process for GSE65904 cohort, and found that *IL27* was also significantly correlated with these tumor-infiltrating immune cells in this cohort as well (Pearson correlation test; *R* = 0.34, *R* = 0.28, *R* = 0.23, respectively; **Supplementary Figures S2D–J**), further highlighting the role of *IL27* in anti-tumor immunity.

The CD8^+^ T cell to regulatory T cell (CD8/Treg) ratio is predictive of the therapeutic efficacy of the immunotherapy (43). Herein, we also found that *IL27* was indeed positively correlated with the CD8/Treg ratio (Pearson correlation test; *R* = 0.14, *P* = 0.02; **Figure 5F**), suggesting that *IL27* was implicated in the immunotherapeutic efficacy.

### *IL27* Could Induce CD8^+^ T Cell Infiltration Through Inhibition of β-Catenin Signaling

**Figure 6:**
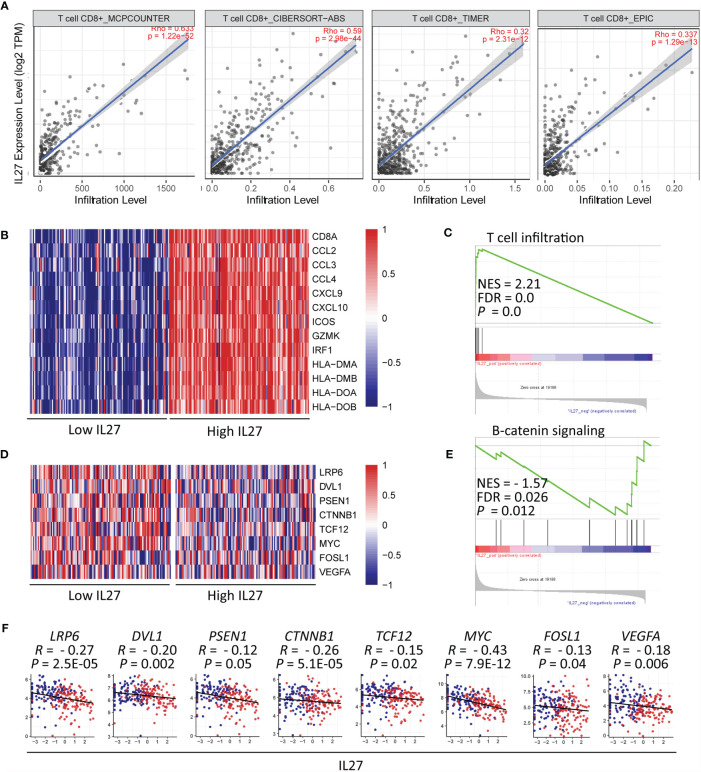
*IL27* might induce CD8^+^ T cell infiltration through inhibition of β-catenin signaling **(A)** *IL27* was positively correlated with CD8^+^ T cell on the basis of TIMER database. **(B)** Heatmap plot showed T cell signature genes were enriched in patients with high levels of *IL27*. **(C)** GSEA results showed *IL27* was positively associated with T cell infiltration. **(D)** The components in β-catenin signaling were enriched in patients with low levels of *IL27.* **(E)** GSEA results showed *IL27* was negatively associated with β-catenin signaling. **(F)** *IL27* was also inversely correlated with components of β-catenin signaling (Pearman’s correlation test).

Considering that CD8^+^ T cells can directly eliminate tumor cells, we decided to further investigate the relationship between *IL27* and CD8^+^ T cells. To confirm the accuracy of our above-mentioned findings, we examined the association between *IL27* expression and CD8^+^ T cell infiltration using TIMER database, and determined that *IL27* was indeed positively correlated with CD8^+^ T cells (**Figure 6A**).

Moreover, we reexamined the role of *IL27* expression on T cell infiltration using GSEA based on RNA-seq data of melanoma patients from the TCGA cohort. The results showed that T cell signature genes were enriched in patients with high levels of *IL27* (**Figure 6B**), and that *IL27* was positively associated with T cell infiltration (FDR = 0.0, NES = 2.21; **Figure 6C**).

To investigate the molecular mechanism of effect of *IL27* on T cell infiltration, we analyzed the relationship between *IL27* expression and β-catenin signaling using GSEA. β-catenin signaling is reported to inhibit T cell infiltration in the TME (44). The findings revealed that genes involved in β-catenin signaling were enriched in patients with low levels of *IL27* (**Figure 6D**), and *IL27* was inversely correlated with β-catenin signaling pathway (FDR = 0.026, NES = -1.57; **Figure 6E**). Moreover, to validate the effects of *IL27* on β-catenin signaling, we analyzed the relationship of *IL27* with the main upstream and target molecules of the catenin signaling pathway, including (*LRP6*, *DVL1*, *PSEN1*, *CTNNB1*, *GSK3B*, *APC*, *APC2*, *AXIN1*, *AXIN2*, *TCF12*, *MYC*, *FOSL1* and *VEGFA*) *(* 44, 45), using RNA-seq data from the TCGA cohort of patients with melanoma. In agreement with the GSEA results, *IL27* was also found to be negatively related to the components of β-catenin signaling pathway (Pearson correlation test; *P* < 0.05; **Figure 6F** and **Supplementary Figure S3A–E**), indicating that *IL27* could stimulate CD8^+^ T cell infiltration *via* suppression of β-catenin signaling.

### *IL27* Could Enhance the Therapeutic Efficacy of Immunotherapy

**Figure 7:**
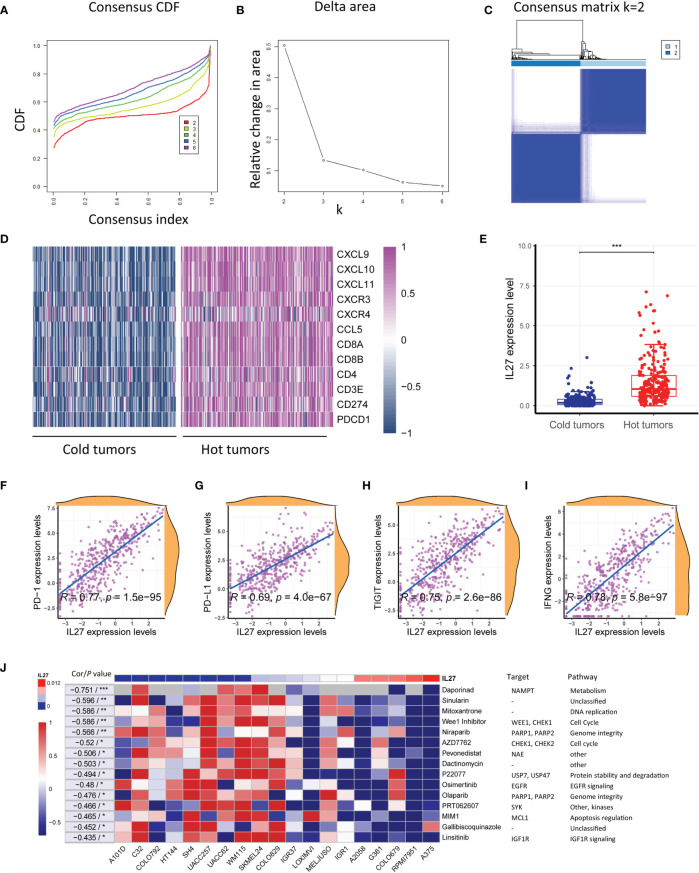
*IL27* was correlated with hot tumor state and improved response to immunotherapy. **(A)** Consensus cumulative distribution functions (CDF) of the consensus matrix for each k (indicated by colors). **(B)** Delta area plot showed the relative change in area under the CDF curve. **(C)** The consensus matrix showed the cluster memberships marked by colored rectangles, enabling a user to figure out a clusters’ member count in the context 38 of their consensus. **(D)** Heatmap plot showed hot tumor signature genes were enriched in hot tumor samples. **(E)** *IL27* was significantly overexpressed in hot tumors, suggesting it was implicated in therapeutic response to immunotherapy. **(F–I)** *IL27* was critically correlated with multiple predictors of response to immunotherapy, including PD-1, PD-L1, TIGIT and IFNG (Pearman’s correlation test). **(J)** Heatmap plot revealed that high expression of *IL27* was associated with low IC50 values in multiple cell lines, further supporting *IL27* could enhance therapeutic response to immunotherapy. *P < 0.05, **P < 0.01, ***P < 0.001.

As the above results suggested that *IL27* was associated with antitumor immunity and might be implicated in response to immunotherapy, we investigated the effects of *IL27* expression on response to immunotherapy. As mentioned earlier, solid tumors can be classified into hot tumor and cold tumors, and hot tumors are responsive to cancer immunotherapy (10). First, we classified 472 melanoma samples from the TCGA cohort into hot tumor samples and cold tumor samples using an unsupervised clustering method on the basis of hot tumor signature genes (*CCL5, CD8A, PDCD1, CD8B, CXCR3, CXCL9, CXCL10, CD4, CD3E, CXCL11, CD274*, and *CXCR4*; **Figures 7A–D** and **Supplementary Table S6**). We then compared *IL27* expression between hot and cold tumors, and observed that *IL27* was significantly overexpressed in hot tumors (*t*-test; *P* < 0.001; **Figure 7E**), suggesting that it was implicated in the therapeutic response to immunotherapy. Consistent with the findings generated from the TCGA cohort, we also performed the same analysis process for GSE65904 dataset (**Supplementary Figures S4A–C**), and observed that *IL27* was markedly upregulated in hot tumors when compared with cold tumors (*t*-test; *P* < 0.001; **Supplementary Figure S4D**), further highlighting the effects of *IL27* on the response to immunotherapy.

To further investigate the therapeutic value of *IL27*, we examined the association of *IL27* with multiple predictors of response to immunotherapy, including *PD-1*, *PD-L1*, *TIGIT* and *IFNG* (41, 42) by analyzing RNA-seq data from the TCGA cohort. Consistent with the previous results, *IL27* was found to be critically correlated with these molecules, with a correlation efficiency ranging from 0.69 to 0.78 (Pearson correlation test; *P* < 0.0001; **Figures 7F-I**). These results strongly suggested that *IL27* might stimulate therapeutic efficacy in cancer treatment.

To reexamine the effects of *IL27* on therapeutic responses, we investigated the association of *IL27* expression with the IC50 of a vast range of agents in various melanoma cell lines using data from CTRP and CCLE. Intriguingly, high *IL27* expression was associated with low IC50 in multiple cell lines, further supporting that *IL27* can enhance the therapeutic response to immunotherapy (**Figure 7J**).

## Discussion

In this study, we revealed that *IL27* had a substantial clinical effect and could serve as an independent predictor of survival outcomes. Further analysis suggested that *IL27* was implicated in TME and programmed cell death including pyroptosis, autophagy and apoptosis in melanoma. Moreover, we revealed that *IL27* was associated with higher T cell infiltration, hot tumor states, cytotoxic molecules and corresponding better therapeutic efficacy. Mechanistically, we speculated that *IL27* might induce CD8^+^ T cell infiltration by suppressing β-catenin signaling, thus enhancing the therapeutic response to immunotherapy.

First, we found that *IL27* had a critical clinical relevance, and could serve as a predictor of survival in patients with melanoma. Specifically, *IL27* was associated with Breslow depth and response to radiotherapy. Radiotherapy has been reported to have a beneficial role in converting immunologically cold tumors into hot tumors (46). Thus, our results strongly suggested *IL27* can play a role in distinct hot/cold tumor states and the resulting therapeutic response, which was confirmed by subsequent analysis.

Another peculiar finding of this study is that *IL27* was found to be primarily involved in pro-tumor immunity, including natural killer cell-mediated cytotoxicity, Toll-like receptor signaling pathway, T cell receptor signaling pathway, NOD-like receptor signaling pathway, RIG-I-like receptor signaling pathway, JAK-STAT signaling pathway, and apoptosis. Further analysis demonstrated that *IL27* was indeed correlated with pyroptosis, autophagy, and apoptosis. Pyproptosis is reported to inhibit the progression of lung cancer, and breast cancer (47–49). Here, we showed the association of *IL27* with pyroptosis, providing evidence of the mechanism underlying *IL27* function in cancer.

We also observed that *IL27* was markedly correlated with immune infiltrates in the TME, including dendritic cells, NK cells, NKT cells, and CD8^+^ T cells. These findings are consistent with those of a previous study (20), supporting the observation that *IL27* can stimulate the immune response in the TME. Mechanistically, we revealed that *IL27* may enhance CD8^+^ T cell infiltration by suppressing β-catenin signaling. Previous studies have reported that activation of β-catenin signaling can repress T cell infiltration into the TME (44). Here, we found that *IL27* was drastically negatively correlated with β-catenin signaling, thus revealing the underlying mechanism of IL27-mediated CD8^+^ T cell infiltration.

In addition, we found that *IL27* could enhance the therapeutic efficacy of immunotherapy for melanoma. *IL27* was reported to drastically enhance the efficacy of immunotherapy without no significant side effects in mouse models of lung cancer (50). Herein, we revealed that *IL27* may stimulate therapeutic efficacy, which was possibly due to two reasons. First, *IL27* could drive effector T cell infiltration and activation, and second, *IL27* could trigger tumor cell pyroptosis, autophagy and apoptosis. In fact, *IL27* can effectively boost both effector immune cells (51) and regulatory immune cells (52). Therefore, the CD8/Treg ratio was considered to be essential for the specific effects of *IL27* in a certain immune context. Consistent with the impact of *IL27* on drug response, we indeed observed that *IL27* expression was positively associated with the CD8/Treg ratio. Together, these findings would provide evidence for the development of cytokine-based immunotherapy in patients who resist the current immunotherapy.

The present study has its own limitations and drawbacks. First, the study was primarily carried out using bioinformatics methods; therefore, laboratory-based experiments are required to support these findings. To compensate for this shortcoming, the main conclusions of the study were confirmed using three or more methods and related literature. For example, the association of *IL27* with immunity was demonstrated by functional annotation, ssGSEA, TIMER, and a correlation analysis with cold/hot tumors. This multi-dimensional verification method has significantly increased the reliability of the results. Second, although this study showed *IL27* could serve as an independent predictor of survival outcomes of melanoma patients from the TCGA cohort, we did not validate this in another cohort due to a lack of such datasets with complete clinical information. Fortunately, the subsequent analysis of the effects of *IL27* on immunity and therapeutic response mechanistically explained that *IL27* can indeed serve as an independent predictor of survival.

In conclusion, *IL27* was considered a predictor of survival outcomes in patients with melanoma. *IL27* expression was shown to drive CD8^+^ T cell infiltration, possibly through suppression of β-catenin signaling, thereby enhancing immunotherapeutic efficacy. These findings will provide important insights for the development of cytokine-based immunotherapy for cancer treatment.

## Data Availability Statement

The datasets presented in this study can be found in online repositories. The names of the repositories and accession numbers can be found in the article/**Supplementary Material**.

## Author Contributions

CZ, ZW, and CD were responsible for the literature review and writing Introduction and Discussion of the manuscript. DD, XZ, and YW analyzed the bioinformatics data and wrote Material and Methods and Results sections of the manuscript. All authors contributed to the article and approved the submitted version.

## Conflict of Interest

The authors declare that the research was conducted in the absence of any commercial or financial relationships that could be construed as a potential conflict of interest.

## Publisher’s Note

All claims expressed in this article are solely those of the authors and do not necessarily represent those of their affiliated organizations, or those of the publisher, the editors and the reviewers. Any product that may be evaluated in this article, or claim that may be made by its manufacturer, is not guaranteed or endorsed by the publisher.

## Supplementary Material

Supplementary Figure 1 Effects of *IL27* on biological processes of melanoma in GSE65904 **(A–C)** Bubble plots demonstrated the top 10 BP, MF and CC terms that were significantly associated with *IL27*, and they were all immune-related. **(D)** Bar plot confirmed the top 10 KEGG terms that were significantly correlated with *IL27*, and they were also immune-related. **(E–G)** *IL27* expression was positively associated with pyroptosis, whereas has nothing to do with autophagy and apoptosis in GSE65904.

Supplementary Figure 2 Association of *IL27* with tumor immunity. **(A–C)** *IL27* was markedly positively correlated with IFNG, GZMB, and immune score, suggesting its role in antitumor immunity. **(D–J)** *IL27* was markedly associated with antitumor immune cells, including gamma delta T cell, natural killer cell, natural killer T cell, dendritic cell, activated CD4^+^ T cell, activated CD8^+^ T cell, and effector memory CD8^+^ T cell.

Supplementary Figure 3 Association of *IL27* with components of β-catenin signaling pathway. **(A–E)** *IL27* was also inversely correlated with components of β-catenin signaling pathway.

Supplementary Figure 4 Association of *IL27* with hot/cold tumor states. **(A)** Consensus cumulative distribution functions (CDF) of the consensus matrix for each k (indicated by colors). **(B)** Delta area plot showed the relative change in area under the CDF curve. **(C)** The consensus matrix showed the cluster memberships marked by colored rectangles, enabling a user to figure out a clusters’ member count in the context 38 of their consensus. **(D)** *IL27* was significantly overexpressed in hot tumors, suggesting it was implicated in therapeutic response to immunotherapy.
