# Supplementary figures and images for: Integrative Characterization of the Role of IL27 In Melanoma Using Bioinformatics Analysis
Source: Front Immunol. 2021 Oct 18;12:713001. doi: 10.3389/fimmu.2021.713001 (PMC8558420; doi:10.3389/fimmu.2021.713001)

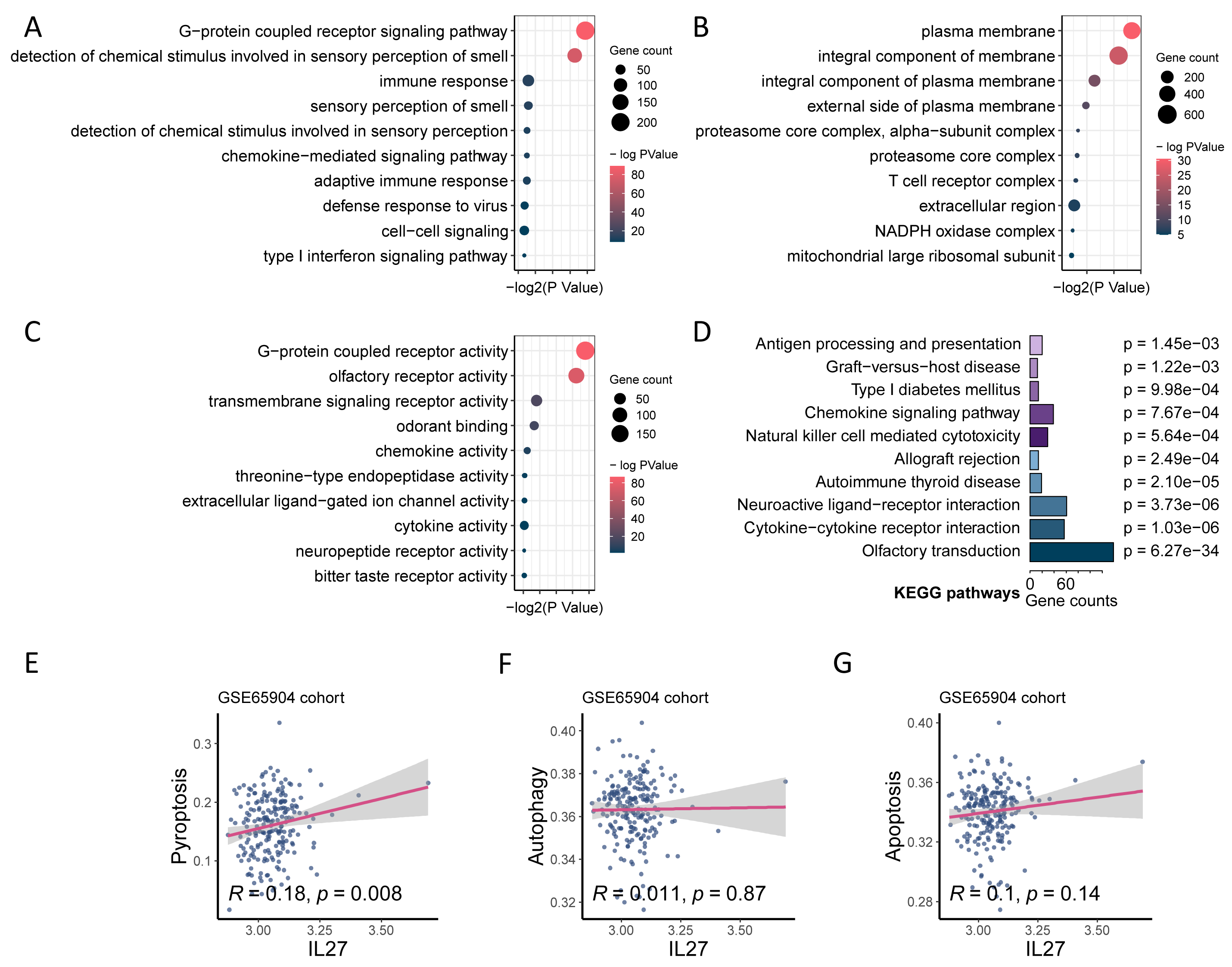

Supplement: Supplementary Figure 1 — Effects of IL27 on biological processes of melanoma in GSE65904 (A–C) Bubble plots demonstrated the top 10 BP, MF and CC terms that were significantly associated with IL27, and they were all immune-related. (D) Bar plot confirmed the top 10 KEGG terms that were significantly correlated with IL27, and they were also immune-related. (E–G) IL27 expression was positively associated with pyroptosis, whereas has nothing to do with autophagy and apoptosis in GSE65904. [file Image_1.tif]

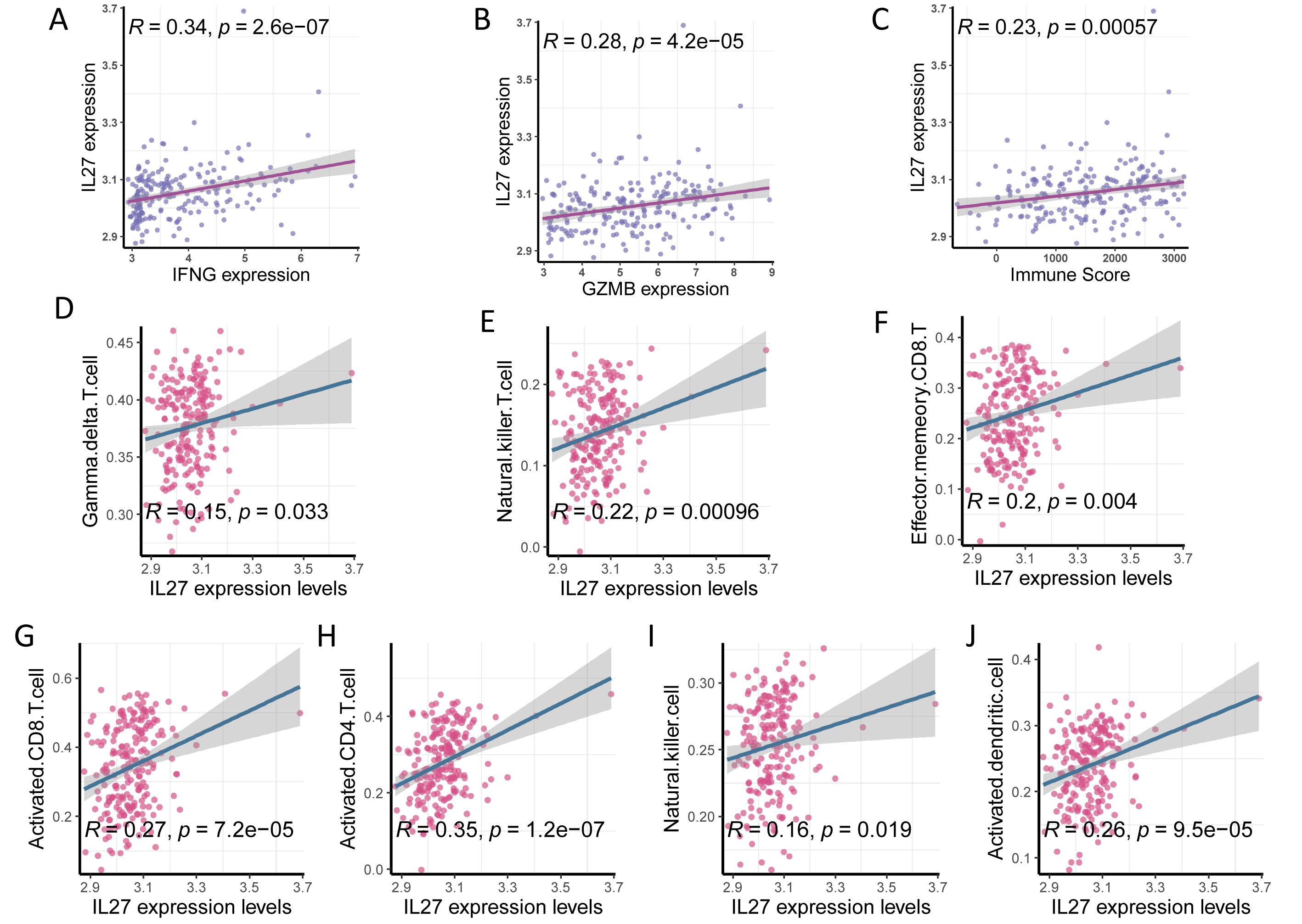

Supplement: Supplementary Figure 2 — Association of IL27 with tumor immunity. (A–C) IL27 was markedly positively correlated with IFNG, GZMB, and immune score, suggesting its role in antitumor immunity. (D–J) IL27 was markedly associated with antitumor immune cells, including gamma delta T cell, natural killer cell, natural killer T cell, dendritic cell, activated CD4+ T cell, activated CD8+ T cell, and effector memory CD8+ T cell. [file Image_2.tif]

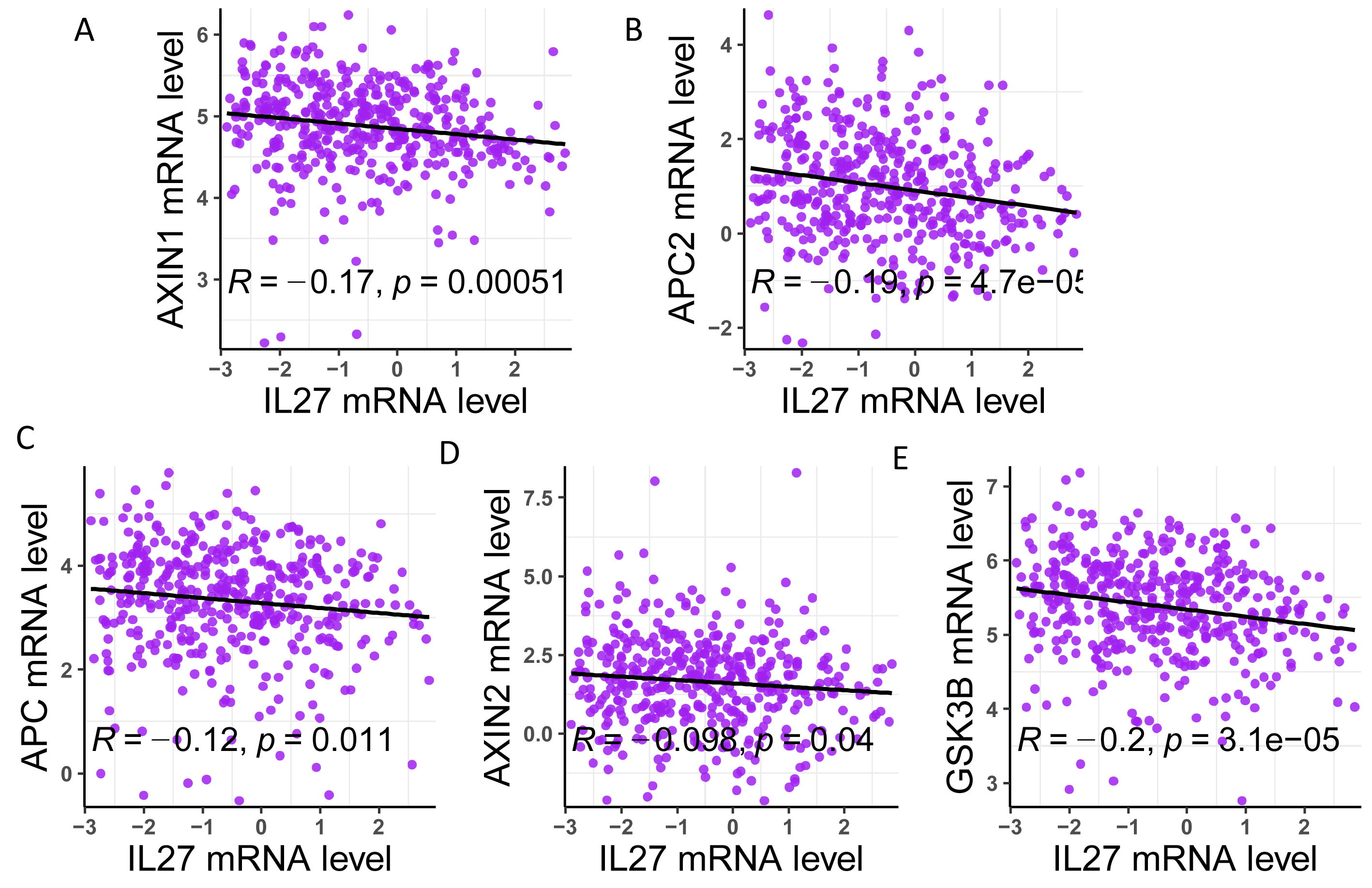

Supplement: Supplementary Figure 3 — Association of IL27 with components of β-catenin signaling pathway. (A–E) IL27 was also inversely correlated with components of β-catenin signaling pathway. [file Image_3.tif]

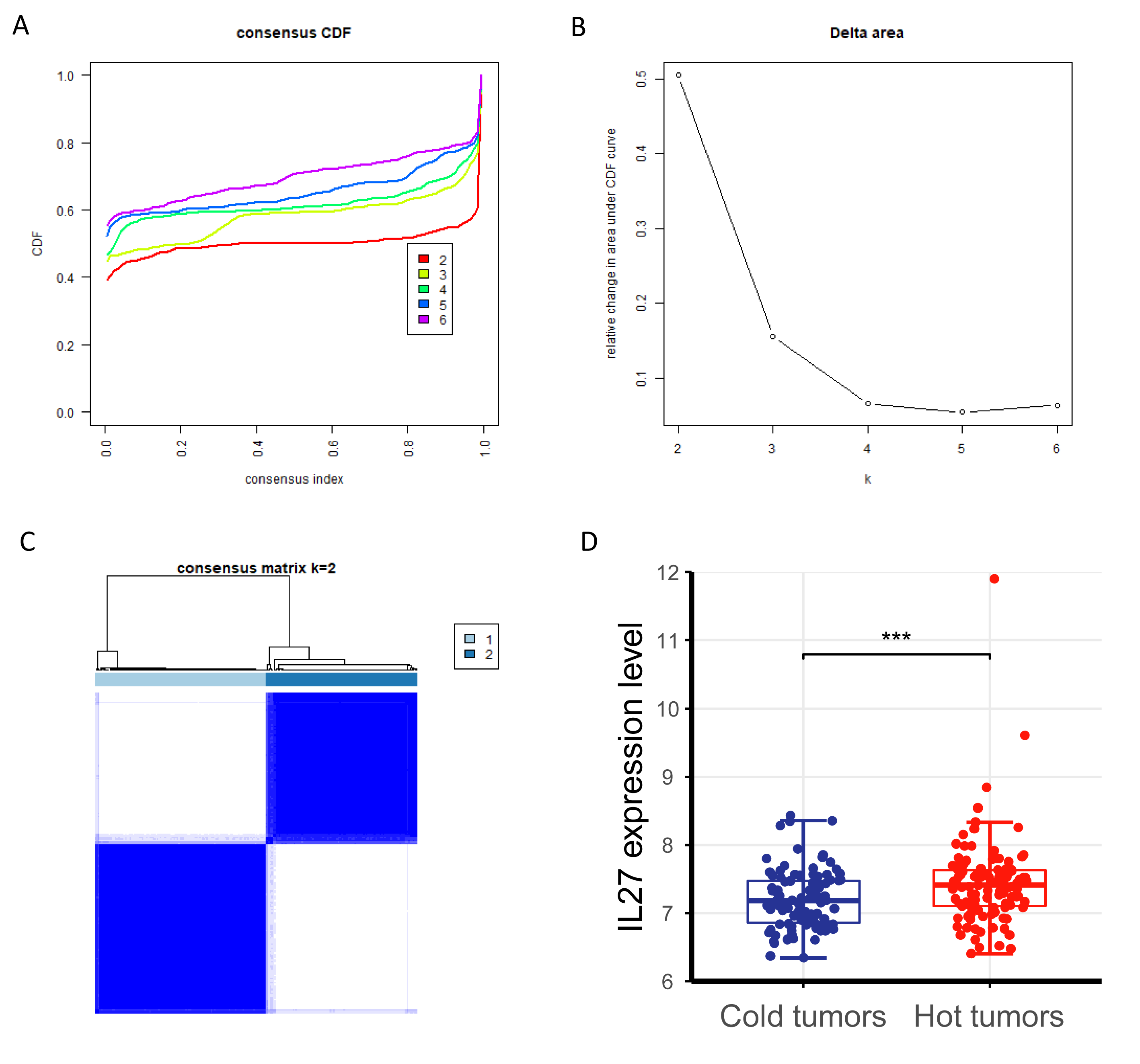

Supplement: Supplementary Figure 4 — Association of IL27 with hot/cold tumor states. (A) Consensus cumulative distribution functions (CDF) of the consensus matrix for each k (indicated by colors). (B) Delta area plot showed the relative change in area under the CDF curve. (C) The consensus matrix showed the cluster memberships marked by colored rectangles, enabling a user to figure out a clusters’ member count in the context 38 of their consensus. (D) IL27 was significantly overexpressed in hot tumors, suggesting it was implicated in therapeutic response to immunotherapy. [file Image_4.tif]
